# Obstacles to the spread of unintuitive beliefs

**DOI:** 10.1017/ehs.2019.10

**Published:** 2019-10-14

**Authors:** Hugo Mercier, Yoshimasa Majima, Nicolas Claidière, Jessica Léone

**Affiliations:** 1 Institut Jean Nicod, Département d’études cognitives, ENS, EHESS, PSL University, CNRS, Paris France; 2 Hokusei Gakuen University, Sapporo, Japan; 3 Aix Marseille Université, CNRS, LPC UMR 7290, 13331, Marseille, France; 4 Institut des Sciences Cognitives Marc Jeannerod, UMR 5304, CNRS and Université de Lyon, Bron, France

**Keywords:** Unintuitive beliefs, cultural evolution, description–experience gap, argument from expertise, deference

## Abstract

Many socially significant beliefs are unintuitive, from the harmlessness of GMOs to the efficacy of vaccination, and they are acquired via deference toward individuals who are more confident, more competent or a majority. In the two-step flow model of communication, a first group of individuals acquires some beliefs through deference and then spreads these beliefs more broadly. Ideally, these individuals should be able to explain why they deferred to a given source – to provide arguments from expertise – and others should find these arguments convincing. We test these requirements using a perceptual task with participants from the US and Japan. In Experiment 1, participants were provided with first-hand evidence that they should defer to an expert, leading a majority of participants to adopt the expert's answer. However, when attempting to pass on this answer, only a minority of those participants used arguments from expertise. In Experiment 2, participants receive an argument from expertise describing the expert's competence, instead of witnessing it first-hand. This leads to a significant drop in deference compared with Experiment 1. These experiments highlight significant obstacles to the transmission of unintuitive beliefs.

## Graphical abstract

**Figure:**
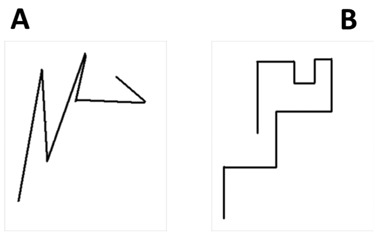

**Media summary:** Accepting unintuitive beliefs is hard. Transmitting them further is even harder.

Many socially beneficial beliefs are unintuitive. Vaccination involves injecting what can be perceived to be a disease into a healthy individual, often a baby, rendering the practice unintuitive (Miton and Mercier 2015). GMOs are perceived as unnatural violations of the essence of natural species, making acceptance of their harmlessness and usefulness unintuitive (Blancke *et al.* 2015). Evolution by natural selection also runs aground on our intuitions about species essences and teleology, making it an unintuitive theory (Shtulman 2006; Shtulman and Valcarcel 2012).

Most efforts dedicated to spreading such unintuitive beliefs have focused on the first step of transmission, for instance by designing pro-vaccination messages to be transmitted by health authorities (e.g. Horne *et al.* 2015). However, studies in communication and political science have revealed the importance of further transmission episodes. In two-step flow model of influence, some members of the public are better informed, in large part thanks to deference toward some sources (media, scientists, etc.), and they are able to influence others in turn (Katz 1957; Katz and Lazarsfeld 1955; see also, Carlson 2018, 2019; Lupia and McCubbins 1998).

For a belief to be transmitted through a two-step flow, not only must some individuals accept the belief (first step), but, in order to complete the second step, (a) these individuals must be motivated to transmit the belief further, along with good reasons to accept it, and (b) other individuals must find these reasons good enough to change their minds. After some background on the transmission of unintuitive beliefs, we explore obstacles that can make it difficult for these two requirements to be met, and then introduce two experiments that test for the presence of these obstacles.

## Accepting unintuitive beliefs

Given the well-known risks entailed by communication – being lied to, manipulated – and the importance of communication for humans, we expect humans to be endowed with mechanisms aimed at evaluating communicated information (Mercier, 2017, in press; Sperber *et al.* 2010). These mechanisms of epistemic vigilance process different cues related to the content and the source of messages in order to decide how much weight to put on them.

One of the most basic epistemic vigilance mechanisms, dubbed plausibility checking, compares communicated information with preexisting beliefs: the more discordant communicated information is with our preexisting beliefs, the more likely the former is to be rejected (for some experimental demonstrations, see Harries *et al.* 2004; Lane and Harris 2015; Yaniv and Kleinberger 2000; for convergent theoretical predictions, see Jaynes 2003; Thagard 2005). This means that, as a rule, unintuitive beliefs face significant difficulties in being accepted.

Some epistemic vigilance mechanisms can overcome the negative reaction to beliefs that challenge our priors, so that people accept unintuitive beliefs. The first is argumentation: good arguments can make people realize that a given conclusion, which they had deemed implausible, is in fact consistent with information they already had or are willing to accept on trust (Laughlin 2011; Trouche *et al.* 2014). Experiments have shown that argumentation enables the spread of unintuitive beliefs in medium-sized discussion groups, as well as along transmission chains (Claidière *et al.* 2017). In these experiments, the arguments relied on mutually agreed upon information – the premises of a reasoning problem – so that no trust was involved. However, the spread of nearly all practically relevant beliefs requires some trust (e.g. that the scientists who conducted the relevant studies about vaccines did not falsify their data).

Trust is the second mechanism of epistemic vigilance that enables the acceptance of unintuitive beliefs. In particular, people defer to other individuals, and can accept unintuitive beliefs, when these other individuals are either *more confident* than they are (e.g. Bahrami *et al.* 2010; Brosseau-Liard and Poulin-Dubois 2014), *more competent* than they are (e.g. Clément *et al.* 2004; Harvey and Fischer 1997) or *belong to a majority* (e.g. Morgan *et al.* 2012, 2015; for reviews, see Clément 2010; Harris 2012; Harris and Lane 2014; Mercier, 2017, in press). However, these experiments focus on a single step: one individual changing their mind by deferring to another (or a group of others). These studies have not examined whether the unintuitive beliefs acquired through deference can then be successfully transmitted to other individuals.

A recent exception is an experiment by Moussaid *et al.* (2017) in which participants solved simple visual tasks. The answers of each participant from a first generation were shown to one participant of a second generation, whose answers were shown to a participant of a third generation, in linear chains six participants long. At each step, the participants could use their own intuitions, as well as the answers of the participant from the previous generation, to form an opinion. Since participants were shown the performance of the previous participant on a series of similar visual tasks, they could detect how competent the previous participant was, and put more weight on the answers of better performing previous participants. This allowed the better performing participants to exert some influence on the participants who received their answers. However, this influence vanished after at most three steps, as deference toward better-performing participants was dampened at each step by the overweighing of the participants’ prior beliefs (the three steps limit has been observed in other areas of social influence, see e.g. Christakis and Fowler 2013). The Moussaid *et al.* (2017) study thus suggests that the cumulative effects of plausibility checking create significant obstacles to the spread of unintuitive beliefs, even when well-founded trust is initially present.

One limitation of the Moussaid *et al.* (2017) experiment is that participants could not communicate with each other: they only saw the answers of the previous participants. While this constraint is often found in cultural transmission studies (Miton and Charbonneau 2018), experiments have shown that allowing participants to communicate can significantly influence cultural transmission (Caldwell and Millen 2009). Moreover, in the standard two-step flow model, people are expected to be able to discuss issues with each other, allowing in particular those who have acquired a belief through deference to explain and justify this deference, thus increasing the chances that the belief is transmitted. Still, as we will see presently, even if people are provided with such an opportunity, they might not seize it.

## First requirement: motivation to transmit arguments from expertise

If an unintuitive belief has been acquired through deference, one way in which it can be transmitted further is by passing on the grounds for this deference. For example, someone who has acquired a piece of news from the *New York Times* could convince someone else to accept it by mentioning that she had read it in the *New York Times*, that is, by making an argument from expertise. As such arguments are easily transmissible, they are especially important not only to bolster two-step effect, but also to extend them, potentially removing the three steps limit previously observed. For instance, logical arguments can extend chains virtually ad infinitum, since they are recreated at each step with no loss (Claidière *et al.* 2017).

However, results show that participants are reluctant, in some situations, to offer arguments from expertise. In one of the studies of Claidière *et al.* (2017), participants were made to accept the unintuitive answer to a reasoning problem through deference, as they were told by the experimenters that this was the correct answer. When these participants were asked to convince another participant in turn, none of them mentioned that they had deferred to an expert, making up bad arguments instead.

One possible reason for people failing to offer arguments from expertise is that they engage in reputation management (see, e.g. Goffman 1959; Leary 1995). More specifically, people might attempt to take credit for their beliefs by minimizing the influence others had in their acquisition (Altay and Mercier, 2019; Shaw and Olson 2015; Silver and Shaw 2018). This is for instance what happens when group members take more credit than they deserve for the positive outcome of a group effort (Schroeder *et al.* 2016).

The reluctance to offer arguments from expertise might be modulated by culture. Even though humans in all cultures seek to self-enhance (Sedikides *et al.* 2003), they do so in different ways. Among US participants, typically very individualistic, reputation management might be best served by appearing more self-reliant. In contrast, other cultures might value reliance on others more. In this respect, Japanese participants are particularly relevant. Japanese participants have been shown to self-enhance on collectivistic attributes (e.g. being a good listener), while American participants self-enhance on individualist attributes (e.g. being self-reliant) (Sedikides *et al.* 2003, 2005).

## Second requirement: accepting arguments from expertise

Even if participants who have accepted an unintuitive belief through witnessing expertise first-hand are motivated to produce an argument from expertise, the argument might not prove as convincing as witnessing expertise first-hand.

Mechanisms of epistemic vigilance process a variety of cues about the sources of messages to decide on how much deference is owed a given source. For example, in the experiment of Moussaid *et al.* (2017), participants could see whether the participant whose answers they received got previous answers right or wrong, and thus how competent that participant was. Such a direct demonstration of competence should be easily processed by epistemic vigilance mechanisms. In contrast, a formal description of the same cue – e.g. ‘this participant got 8 out of 10 answers correct’ might not trigger mechanisms of epistemic vigilance in the same way.

Differences between *decisions from experience* and *decisions from descriptions* have been observed in several domains (Hertwig and Erev 2009; Hertwig *et al.*, 2004; Shlomi 2014). For example, when it comes to deference toward majority opinions, it seems that participants are able to process cues from experience – seeing the converging answers of several individuals – nearly optimally (e.g. Morgan *et al.* 2012). In contrast, when similar information is presented as a description (e.g. ‘80% of the group holds such and such opinion’), participants do not properly weigh the majority opinion (Mutz 1998; for review, see Mercier and Morin 2019).

## The current experiments

In order to experimentally test the transmission of unintuitive beliefs in a two-step flow model, we designed an experimental setup in which participants acquire an unintuitive belief through well-founded deference, and are then asked to convince another participant to accept this belief. In Claidière *et al.* (2017), participants had been observed to use (poor) logical arguments to transmit an answer to a logical problem they had accepted through deference to expertise. In order to make it as difficult as possible for participants to use this strategy, we used here not a logical problem, but a perceptual task: to tell which of two lines forming squiggles is the longest. Our goal was to create a situation in which participants would be prompted to use arguments from expertise: the grounds for deference were transparent, the transmission took place right after the new belief had been acquired so that memory loss was not an issue, and other good arguments were not obvious to find. As a result, if we observe difficulties in the transmission of arguments from expertise in this paradigm, this bodes poorly for contexts in which other obstacles might be present.

After a pilot study, which established that our materials have the desired properties, Experiment 1 focused on the first requirement of the two-step flow model. This experiment was inspired by Moussaid *et al.* (2017). As in the second generation of Moussaid *et al.* (2017), our participants solved a perceptual task, were shown the answer of one (or, in the present experiment, several) previous participant(s), and could take this input into account in their decision. The answers the participants received were in fact created by us in such a way that the participants should be deferent toward this previous (supposed) participant. Participants then passed on one of their answers to another participant but, unlike in Moussaid *et al.* (2017), our participants were provided with the opportunity to defend this answer with an argument aimed at convincing the next participant. The main outcome of Experiment 1 is the participants’ propensity to produce arguments from expertise.

In Experiment 2, participants were in a situation similar to that of the participants of Experiment 1 but, instead of witnessing first-hand someone else's expertise, they received an argument from expertise. This represents the second requirement for the two-step flow model to be completed. We measured whether these argument from expertise changed participants’ minds, in particular whether they were as potent as witnessing the expertise first-hand (as participants did in Experiment 1).

In both experiments we induced deference toward the previous (supposed) participant in three ways, representing the main mechanisms of deference: high confidence, competence and majority. We did not make predictions about differences between these conditions, using them to increase the breadth of our study and the robustness of our results.

To test for potential effects of culture, both experiments were conducted with participants in the US and in Japan. Besides specific cultural differences in how reputation is managed (see above), US and Japanese participants differ in a number of relevant ways. Japanese participants tend to be less overconfident than US participants (Yates *et al.* 1998), which might influence the type of arguments they generate. Studies also suggest that Japanese participants have lower interpersonal trust than US participants (Miller and Mitamura 2003; Yamagishi 2001; Yamagishi and Yamagishi 1994), which might make them less likely to change their minds when presented with arguments that require the premises to be taken on trust. Still, in spite of these potential cross-cultural differences, our main objective was to uncover cross-culturally robust constraints on the transmission of unintuitive beliefs.

## Pilot study

Experiment 1 relied on getting participants to accept an unintuitive answer on the basis of deference, to test whether they were then motivated to produce arguments from expertise in order to further transmit this unintuitive answer. Accordingly, the pilot study had two goals: (a) to check that, on the crucial question, most participants initially provide the intuitive but incorrect answer; and (b) to check that, on the crucial question, most of the participants who initially provided the intuitive answer accepted the unintuitive but correct answer. This pilot study was only conducted among US participants, as we did not expect significant cultural differences at that level (Experiment 1 suggests that this was a reasonable expectation, but for one condition).

## Method

### Participants

A total of 151 participants were recruited online using Amazon Mechanical Turk (73 females, *M*_age_ = 25.0, SD = 17.5). They were paid (at least, see below for bonuses) $0.50 for their participation and had to be located in the US.

### Materials

**Figure 1.**
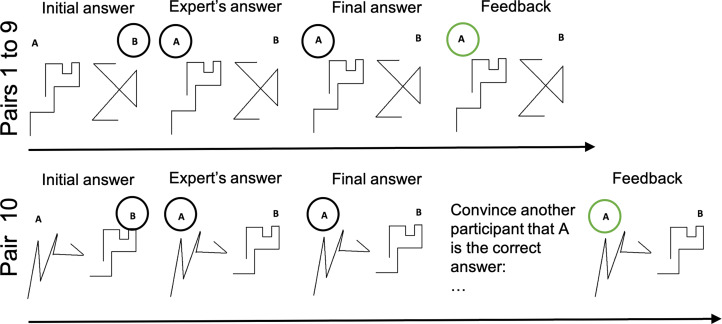
Procedure for Experiments 1 and 2 (in the pilot study, the tenth pair was not treated differently from the other pairs). On pairs 1–9, participants have to tell which of two lines is the longest (initial answer). They are then provided with the answer of (supposed) previous participant(s) (expert's answer); they can change their mind on the basis of this answer (Final answer), and they are provided with feedback on the accurate answer. Pair 10 is identical to the first nine except that, before they receive feedback, participants are asked to formulate an argument to convince someone to accept their final answer (figure under CC-BY-SA).

We used material that was known to yield intuitive but incorrect answers. Participants saw pairs of lines, and they had to tell which line was the longest. For some pairs, previous experiments had revealed a strong tendency to select one of the two lines. Sometimes this intuitive answer is incorrect (two examples are provided in Figure 1). We thank Asher Koriat for kindly providing us with this material. More information on how the pairings were created can be found in Koriat (2011). As in Koriat (2011), we used some of the lines twice, but never the same pairs.

### Procedure

Participants had to compare 10 pairs of lines. Based on previous results (Koriat 2011), we used five easy pairs (i.e. most participants tended to provide the correct answer), and five difficult pairs (i.e. most participants tended to provide the wrong answer) (see the Electronic Supplementary Materials – ESM – for the full stimuli set). After they had provided an initial answer, participants were told of the answers given by (what they thought to be) another participant (or participants) in a previous experiment. They could change their mind on the basis of this information. Participants then received feedback on whether they, as well as the other participants, had provided the correct answer. Since the answers provided to the participants were designed to indicate a high degree of competence, we call the (pretend) participants who provide them the experts. However, remember that this expert status is gained purely by observation of good performance, while being described to the participants as being another participant just like them. The first line of Figure 1 represents the four stages associated with each pair of lines (including the tenth) in the pilot study.

Participants were distributed in three different conditions, as a function of the type of answers described as coming from previous participants that they received (these answers were all created by us). In the competence condition, participants received the answers from a (supposed) participant who was always correct.

In the confidence condition, participants had to indicate their degree of confidence in their answer, and they received information from the (supposed) participant in the form of an answer and a degree of confidence (expressed verbally) for each pair. The answers they received were not always right, but they were always well calibrated: on the first nine questions, six answers were correct and expressed with strong confidence, while three were incorrect and expressed with low confidence. The answer on the tenth question was expressed with strong confidence.

Finally, in the majority condition, participants received the answers from seven (supposed) participants. For each question, a majority (of five for seven questions and four for two questions) supported the correct answer. The previous (supposed) participants were identified with numbers, and they each made at least two mistakes before reaching question 10. On the tenth question, six out of the seven previous (supposed) participants offered the same answer.

We aimed at creating conditions in which participants would have received, when they reached question 10, strong cues that they should follow the opinion of the previous (supposed) participant. To maximize the chances that the participants would pay attention to this information, they were told that they would receive a bonus ($0.50) if they got nine or 10 of the answers correct.

## Results and discussion

This pilot had two main goals. The first was to check whether most participants failed on difficult pairs, and succeeded on easy pairs. Summing over the three conditions, all pairs gave the expected results, with at least half (77% overall) of the participants providing the correct answer on easy pairs, and at least half (69% overall) of the participants providing the incorrect answer on difficult pairs. Crucially, a majority of participants (71%) provided the incorrect answer on the tenth pair, critical for the following experiments.

The second goal was to check whether a sufficient share of participants who had provided the intuitive but wrong answer on the tenth pair changed their minds on the basis of the expert's answer. This tenth pair was crucial since it is the answer to that pair that participants had to attempt to convince another participant of in Experiment 1. In each condition, at least half of the participants who had provided an incorrect initial answer changed their minds (competence condition, 82%; confidence condition, 58%; consensus condition, 80%; average, 74%).

## Experiment 1

The pilot study established that most participants from a US population initially provided the wrong answer on the tenth pair, and that most of the participants who provided the wrong answer changed their mind when presented with the answer of a source they should defer to. The goal of Experiment 1 was to test the type of arguments people provided in an attempt to transmit this answer to another participant: if they did not provide arguments that described the expertise, the second step of the two-step flow model would be jeopardized (as well as any further step). Before participants received feedback on the accuracy of their answer to the tenth question, we asked them to provide an argument in order to convince another participant.

## Method

### Participants

For the US, 299 participants were recruited online using Amazon Mechanical Turk (158 females, *M*_age_ = 35.9, SD = 11.0). They were paid at least (see below for information on bonuses) $0.30 for their participation and had to be located in the US. For Japan, 304 participants were recruited online using a Japanese crowdsourcing tool CrowdWorks (204 females, *M*_age_ = 34.6, SD = 8.3). They were paid at least JP¥ 35 for their participation and had to be located in Japan. The sample size was based on two factors. The first was an estimate, grounded in the pilot results, of what share of participants would change their minds on the basis of the previous participant's answer – since it is primarily these participants we are interested in. The second was an estimate of the size of the first-hand (Experiment 1) vs second-hand (Experiment 2) effect. The sample size chosen should allow us to detect at least moderate effects.

### Materials

The materials are identical to those of the pilot study.

### Procedure

The procedure was identical to that of the pilot study, with one exception. On the tenth pair of lines, before they received feedback on the accuracy of their final answer, the participants were told ‘Your answer to this question will be passed on to another participant in a similar experiment in the next few days. You can give this participant an argument for why they should accept your answer’ (see Figure 1).

The three conditions (competence, confidence and consensus) were created so that when the participants reach the tenth pair, the accumulated feedback suggested that they should defer to the expert (i.e. the previous participant(s) whose answers they received). To maximize the chances that the participants would pay attention to this information, they were told that they would receive a bonus ($0.50/JP¥ 60) if they got nine or 10 of the answers correct.

To motivate participants to write convincing arguments, a second bonus (also of $0.50/JP¥ 60) was created. In the Bonus if Right condition, participants received the bonus if the participant their answers were passed to gave the correct answer. In the Bonus if Convinced condition, participants received a bonus if the participant their answers were passed to changed their mind to adopt the answer the participants supported. In both cases, participants were told that the next participant would be aware of this incentive scheme. Assuming participants believed their own answer to be correct, both incentive schemes should motivate them to create convincing arguments. Since preliminary analyses suggested that the type of bonus had no effect on the participants’ behavior, this variable will not be further analyzed.

## Results

In line with the results from the pilot, the majority of participants initially provided the wrong answer on the tenth question (US, 78%, Japan, 87%), and in all conditions except one (the confidence condition for Japanese participants), a majority of these participants changed their minds when exposed to the opinion of the expert (see left-hand side of Table 1). A preliminary analysis of the results in the confidence condition revealed that Japanese participants, unlike US participants, were not more likely to be convinced when the expert was highly confident in their response compared with when she was not confident (over the 10 pairs, 14% changed their mind when the expert was confident, and 11% when they were not), suggesting that the participants did not consider the feedback from the expert as expected. The present work focuses on people who have acquired a belief through deference to reliable cues, such as competence, high confidence or majority. Given that the Japanese participants did not appear to consider high confidence as a reliable cue in the first place, we decided to analyze the confidence condition separately from the other conditions. Combined analyses (which yield broadly similar results) are available in the ESM.

**Table 1.** Percentage of participants who had initially given the wrong answer and were convinced by the expert to accept the correct answer, as a function of the reason to believe the expert (competence, confidence, or consensus), the way the participants encountered the expert (first-hand experience vs. argument from description) and the country in which the experiment took place. Numbers in brackets are 95% confidence intervals obtained from generalized linear models described in the text

Condition	First-hand experience (Experiment 1)		Argument from description (Experiment 2)	
	US	Japan	US	Japan
Competence	66% [55; 76]	64% [53; 74]	48% [37;58]	18% [11; 27]
Confidence	65% [53; 75]	38% [28; 48]	43% [32; 54]	31% [23; 42]
Consensus	79% [70; 87]	79% [70; 86]	50% [40; 60]	18% [11; 28]
Total	71%	60%	47%	23%

Only looking at participants who started out with the wrong answer on the tenth pair, we used a binary variable (convinced or not by the expert) to analyze the effect of condition (competence and consensus). We found no significant interaction between country and experimental condition (generalized linear model (GLM), *β* = −0.09, SE = 0.50, *z* = −0.18, *p* = 0.86). The effect of expertise was similar in Japan and in the US (GLM, *β* = 0.11, SE = 0.33, *z* = 0.33, *p* = 0.74) with the effect of expertise significantly more pronounced in the consensus condition compared with the competence condition (GLM, *β* = 0.75, SE = 0.34, *z* = 2.21, *p* = 0.03).

Regarding confidence, we found a large difference between the US and Japan (GLM, *β* = 1.14, SE = 0.34, *z* = 3.40, *p* < 0.001). Japanese participants were much less likely to be convinced by the confident expert than US participants (see Table 1). This might be explained by the fact that the Japanese participants did not discriminate between high- and low-confidence statements from this expert (see above). In turn, the lack of reliance on confidence in Japan might be related to differences in how Japanese and US participants express confidence, with US participants being usually more overconfident than Japanese participants (Yates *et al.* 1998).

The main outcome of interest here is the types of arguments generated by the participants to support their answer on the tenth question. The arguments were coded according to whether they were arguments from expertise or not. The arguments were coded by one of the authors. Twenty percent of the arguments were then coded by a second coder, blind to the hypotheses, reaching an acceptable level of interrater agreement (*k* = 0.83). This includes the finer grained coding described below.

Arguments from expertise are arguments that include a mention of the information provided to the participant from the various sources (e.g. ‘Previous participants who have gotten most, if not all, questions similar to this have answered the same’ or ‘The person before me was always correct, so I just copied them’).

Focusing on arguments provided by participants who accepted the unintuitive answer through deference to expertise – those most relevant to the spread of unintuitive beliefs – Table 2 breaks down the proportion of arguments from expertise as a function of condition and country.

**Table 2.** Share of arguments from expertise as a function of condition and country, for participants who accepted the correct answer through deference to the expert. The 95% confidence intervals in brackets were calculated from the statistical models described below. The full breakdown of arguments is included in the ESM

Condition	Share of arguments from expertise	
	US	Japan
Competence	29% [19; 43]	37% [25; 51]
Confidence	16% [8; 29]	9% [3; 25]
Consensus	55% [43; 66]	24% [16; 35]
Total	36%	25%

We used a binary variable (argument from expertise or not) to analyze the effect of country and condition (competence and consensus, confidence being kept separate for reasons explained above) on the prevalence of arguments from expertise. We found a significant interaction between country and experimental condition (GLM, *β* = −1.71, SE = 0.56, *z* = −3.072, *p* = 0.002). There was no difference between countries in the transmission of arguments from expertise in the competence condition (GLM, *β* = 0.11, SE = 0.33, *z* = 0.33, *p* = 0.74) but in the consensus condition the odds of having an argument from expertise were 290% higher in the US compared with Japan (GLM, *β* = 1.36, SE = 0.36, *z* = 3.74, *p* < 0.001).

Using the same approach, we found no difference in the proportion of arguments from expertise between the two countries in the confidence condition (GLM, *β* = 0.61, SE = 0.73, *z* = 0.84, *p* = 0.40).

Among the other arguments offered by the participants, one type was particularly interesting: perceptual arguments, that is, arguments based on properties of the lines (e.g. ‘the three longest lines in “A” seem to equal most of the smaller segments in “B”’; for the full breakdown of argument types that were coded, see ESM). Some 24% of participants in the US and 11% in Japan produced such arguments. These participants thus argued that line A looked longer than line B because of some perceptual characteristic of line A even though, when they had initially seen the lines, they had thought that line B was the longest.

## Discussion

As expected, most participants initially provided the wrong answer to the critical question and, among those participants, most accepted the answer provided by the expert (i.e. a previous participant), with the exception of Japanese participants in the confidence condition (this condition was thus analyzed separately). Focusing on the participants who changed their mind to accept the expert's answer, we observed that many failed to mention in their arguments the expert from whom they had derived their answer. Only in one condition, in one country, was there a (slim) majority of arguments from expertise (the consensus condition among US participants: 57%). In contrast, some participants offered perceptual arguments, suggesting that they had been able to answer correctly on their own, simply by looking at the lines.

The fact that only a minority of arguments mentioned the expert who had led participants to change their minds and accept an unintuitive belief jeopardizes the first requirement of the two-step flow model. Not only do only a minority of participants who deferred to an expert mention this expert in their arguments, but, when they do so, their arguments might not prove as convincing as if they had provided first-hand evidence that the expert should be deferred to. In Experiment 2, we test how participants react to such arguments, when they represent fairly accurately the first-hand experience of participants from Experiment 1.

## Experiment 2

Experiment 2 tests the second requirement of the two-step flow model. More specifically, Experiment 2 asks whether good arguments from expertise are as effective as having had first-hand experience of someone's expertise. We created arguments that described, as accurately and convincingly as we thought possible, the experience of participants in Experiment 1. We then gave these arguments to participants who had previously faced the same 10 pairs of lines, so they would be in a situation similar to the participants of Experiment 1, and would easily understand the situation described in the arguments.

## Method

### Participants

For the US, 306 participants were recruited online using Amazon Mechanical Turk (152 females, *M*_age_ = 36.6, SD = 11.9). They were paid at least (see below for information on bonuses) $0.30 for their participation and had to be located in the US. For Japan, 304 participants were recruited online using a Japanese crowdsourcing tool CrowdWorks (172 females, *M*_age_ = 37.3, SD = 9.8). They were paid at least JP¥ 35 for their participation and had to be located in Japan.

### Materials

The materials were the same as in Experiment 1.

### Procedure

The participants answered pairs 1–9 without receiving the opinion of another participant (but with feedback on their own performance). On the tenth pair, after they had provided an initial answer, participants were provided with what was presented as the answer of a previous participant, defended by an argument. This answer was always the correct, unintuitive answer, and the arguments were as follows:

- *Competence condition* – for every one of the 10 problems, I received the answer from another participant. He was right on every one of the first nine questions, even when I thought he'd be wrong. For this question he answered A. Given that he's always been right, I think he'll get it right again.
- *Consensus condition* – for every one of the 10 problems, I got the answers from another seven participants. The majority was right on every one of the first nine questions, even when I thought they'd be wrong. For this question the majority of them answered A. Given that the majority has always been right, I think it'll be right again.
- *Confidence condition* – for every one of the 10 problems, I got the answer from another participant. He was right on most of the first nine questions. Also, when he was wrong, he wasn't really sure, but when he was right, he was really sure every time. For this question he answered A and he was really sure. Given that he's always been right when he was sure, I think he'll get it right again.

Participants were also told that ‘This participant was told that they would receive a bonus if you gave the correct answer’, so that they should have no reason to doubt the veracity of the premise of the argument. To maximize the chances that the participants would pay attention to this information, they were told that they would receive a bonus ($0.50/JP¥ 60) if they got nine or 10 of the answers correct.

## Results

As in Experiment 1, most participants initially provided the wrong answer on the tenth pair (US, 83%; Japan, 82%). Of these participants, the share that changed their minds when confronted with the argument from expertise is presented on the right-hand side of Table 1.

We used a binary variable (convinced or not by expertise) to analyze the results of a three-way interaction between country (US and Japan), experiment (1 and 2) and condition (competence and consensus, confidence being analyzed separately for the same reasons as in Experiment 1: Japanese participants did not respond to the confidence cue). We found no significant interaction between country, experimental condition and experiment (GLM, *β* = 0.16, SE = 0.71, *z* = 0.23, *p* = 0.82). However, the two-way interaction model with country and condition revealed that the gap between experience (i.e. witnessing first-hand the expert's reliability) and description (i.e. being provided with an argument summarizing this information) was much larger in Japan than in the US (GLM, *β* = 1.43, SE = 0.35, *z* = 4.06, *p* < 0.001). In Japan, the odds of being influenced by expertise decreased by an estimated 92% (GLM, *β* = −2.48, SE = 0.27, *z* = −9.37, *p* < 0.001), compared with an estimated 65% for the US (GLM, *β* = −1.04, SE = 0.23, *z* = −4.50, *p* < 0.001).

Regarding confidence, we found no interaction between country and experiment (GLM, *β* = 0.66, SE = 0.46, *z* = 1.43, *p* = 0.15). We found a similar decrease in the influence of expertise between experience and description (GLM, *β* = −0.93, SE = 0.34, *z* = −2.75, *p* = 0.006), but this effect was mostly driven by US participants who were more influenced by confident expertise than the Japanese participants in the first experiment (see Table 1 and reasons for this difference suggested above).

## Discussion

Compared with participants who witnessed first-hand the reliability of the expert (Experiment 1), participants who were provided with an argument from expertise describing this reliability (Experiment 2) were less likely to change their mind and accept the unintuitive belief. This was true, at least descriptively, in each of the three conditions and in both countries. Moreover, the difference between the two information formats was stronger in Japan, where only a small minority of participants accepted the unintuitive belief when it was supported by an argument.

By telling participants that the participant whose argument they were reading had their best interests at heart, we had attempted to eliminate concerns about participants not trusting the premise of the argument provided. However, it is possible that this manipulation did not function perfectly, so that not all participants fully believed the premise. The lower level of acceptance of the unintuitive belief in Experiment 2 in Japan compared with the US would then be compatible with the lower levels of interpersonal trust in Japan, compared with the US (Miller and Mitamura 2003; Yamagishi 2001; Yamagishi and Yamagishi 1994).

## Conclusion

In a two-step flow model of communication, beliefs are acquired by a first group of individuals, who then relay them to the rest of the population. We tested two requirements to the spread of unintuitive beliefs in a two-step flow model: (a) individuals from the first group should provide arguments from expertise to convince others to accept the belief they have acquired; and (b) arguments from expertise should prove persuasive for the rest of the population – ideally, as persuasive, otherwise the chain of influence will quickly stop.

Experiment 1 tested the first requirement: that arguments from expertise be produced. Participants (in the US and Japan) witnessed first-hand the reliability, in a perceptual task, of a previous participant (or participants), whom we call the expert. Deference to this expert led most participants to change their mind on a question, accepting an unintuitive answer (with one exception in the case of a confident expert for Japanese participants). However, when these participants, who owed their belief to deference to the expert, were asked to convince another participant in turn, only a minority mentioned the expert in their arguments. In contrast, some participants used perceptual arguments even though, when they had first been presented with the task, their perception had led them to a different answer.

Experiment 2 tested the second requirement: that arguments from expertise are convincing. Participants were put in a situation similar to that of the participants of Experiment 1 but, instead of witnessing first-hand the reliability of the expert, they were provided with an argument from expertise summarizing the reasons for deferring to the expert. Both in the US and in Japan, these arguments were less likely to change participants’ minds than the first-hand experience of Experiment 1.

Our experiments have several limitations. First, it could be argued that the correct answer to the crucial pair of lines is not very unintuitive – after all, a minority of participants initially provides the unintuitive answer. However, this is also true for other problems in which the wrong answer is commonly thought to be unintuitive (such as the problems of the Cognitive Reflection Test; Frederick 2005). Moreover, if we can already observe obstacles to the spread of unintuitive beliefs in the case at hand, these obstacles should only grow stronger if more unintuitive beliefs are examined.

Another limitation is that, as suggested above, the participants in Experiment 2 might not have trusted entirely the previous participant to tell the truth in the premise of the argument. This would provide another explanation, besides the experience–description gap mentioned in the introduction, for the drop in acceptance of the unintuitive belief in Experiment 2 relative to Experiment 1. Future experiments should attempt to tease out these two factors. However, it should be noted that the drop was observed even though we had provided participants with every reason to trust the premise of the argument (i.e. telling them that the previous participant's interests were aligned with theirs). If mistrust hinders the spread of unintuitive beliefs even in such arguably ideal circumstances, it might form another intrinsic limit to the spread of unintuitive beliefs.

One could also wish that the interaction between the participant who witnessed the expertise first-hand and the next participant be more natural, for instance that the participants have a face-to-face discussion. Experiments have shown that face-to-face discussion is more efficient at changing people's minds than a unidirectional offer of reasons, at least regarding the answers to logical problems (Claidière *et al.* 2017; Trouche *et al.* 2014). In the case at hand, a face-to-face discussion might have prompted more participants to resort to arguments from expertise, or to be more accepting of arguments from expertise. However, preliminary results from an experiment conducted using similar stimuli to the current Experiment 1, but in which participants were able to talk to the next participant, suggest that this is not the case (Castelain *et al.*, 2019). In this experiment (conducted with indigenous populations in Ecuador), participants used very few arguments from expertise, replicating the current results.

Finally, our stimuli are – by design – quite removed from socially relevant unintuitive beliefs, such as a belief in the efficacy of vaccination. One of the pertinent differences is that in the cases of these socially relevant beliefs, expertise can be characterized not merely by good performance, but also by a deeper understanding of the causal mechanisms at play: a doctor can not only cure people, but also explain how a therapy functions. Such explicit causal understanding, if transmitted alongside a correct answer, should help the further transmission of the correct answer. However, in many domains, passing along such explicit causal understanding might prove difficult, as this understanding requires rich background knowledge (e.g. in many cases doctors can only explain to their patients why a therapy is efficient in relatively crude terms). Moreover, in other domains people might perform well enough without much by way of causal understanding (for example, some of the authors of the present article do not have a good causal understanding of what makes a scientific article successful; more generally, see Henrich 2015). As a result, in some cases at least, beliefs acquired through deference to expertise would have to be passed along without any accompanying causal understanding.

Even if our stimuli differ in some ways from socially relevant beliefs, we sometimes observe similar outcomes for both. For example, a study of the arguments provided by pro-vaccine members on an Internet forum showed that the arguments mostly rested on personal experience – the equivalent of the perceptual arguments in the present experiments (Faasse *et al.* 2016; Fadda *et al.* 2015). This is true even though the pro-vaccine members must have believed sufficiently in the efficacy of vaccination – presumably through deference – to have had their children vaccinated in the first place. Further research could attempt to experimentally investigate obstacles to the spread of more socially relevant unintuitive beliefs (see Altay and Mercier, 2019).

In spite of these limitations, our results provide some of the first experimental evidence of significant obstacles in the spread of unintuitive beliefs acquired through deference. Moreover, our studies point to multiple sources for these obstacles, some cognitive (variations in information formats), others motivational (failure to produce arguments from authority, possibly because of reputation management goals), suggesting that to improve the transmission of unintuitive beliefs, multiple problems have to be tackled at once.

In the field of cultural evolution, much attention has been focused on how an individual's success or prestige can prompt others to copy them (e.g. Henrich and Gil-White 2001; Laland 2004; Richerson and Boyd 2005). In contrast, few studies have looked at whether a practice or belief acquired in this way can then be further transmitted. Our results suggest that significant obstacles can emerge in these further steps, so that for success or prestige to explain the wide and persistent spread of cultural elements, other factors must also be at play. For example, the beliefs one has acquired through deference might help make oneself successful or prestigious – as if the participants in our experiment learned not only the correct answer, but also how to find the correct answers in the future (e.g. if sound causal models of the world had been transmitted alongside the correct answer). Another possibility is that the successful or prestigious source is able to provide sound arguments for their beliefs, arguments that others can then use to transmit the beliefs further (as in Claidière *et al.* 2017).

## Data Availability

All of the data are available at https://osf.io/mqeas/

## Supplementary material

For supplementary material accompanying this paper visit http://dx.doi.org/10.1017/ehs.2019.10. click here to view supplementary material
