# Supplementary material for: Obstacles to the spread of unintuitive beliefs
Source: Evol Hum Sci. 2019 Oct 14;1:e10. doi: 10.1017/ehs.2019.10 (PMC10427286; doi:10.1017/ehs.2019.10)
Supplement: Supplementary file 1 [file S2513843X19000100sup001.zip › S2513843X19000100sup001/ESM.docx]

Electronic Supplementary Materials to “Obstacles to the spread of unintuitive beliefs”

Mercier, Hugo; Majima, Yoshimasa; Claidière, Nicolas; Léone, Jessica

**Experiment 1**

*Complementary analysis.*

This analysis presents all the data together, instead of analyzing the Confidence condition separately, as in the main text.

Effect of expertise

Only looking at participants who started out with the wrong answer on the tenth pair, we used a binary variable (convinced or not by the expert) to analyse the effect of condition (Competence, Consensus and Confidence). We found a significant interaction between country and condition. There was no effect of country for competence (GLM, β=0.11, SE=0.33, z=0.33, p=0.74) or consensus (GLM, β=0.02, SE=0.37, z=0.06, p=0.95) but an effect for confidence (GLM, β=1.14, SE=0.34, z=3.40, p=0.001). The effect of expertise was significantly more pronounced in the consensus condition compared to the competence condition (GLM, β=0.75, SE=0.34, z=2.21, p=0.03) and significantly less pronounced in the confidence condition compared to the competence condition in Japan (GLM, β=-1.08, SE=0.32, z=-3.36, p=0.001) but not in the US (GLM, β=-0.05, SE=0.35, z=-0.13, p=0.90).

Type of arguments

We used a binary variable (argument from expertise or not) to analyse the effect of country and condition (competence, consensus and confidence) on the prevalence of arguments from expertise. We found a significant interaction between country and experimental condition. There was no effect of country for competence (GLM, β=0.35, SE=0.42, z=0.84, p=0.40) or confidence (GLM, β=-0.61, SE=0.73, z=-0.84, p=0.40) but an effect for consensus (GLM, β=-1.36, SE=0.36, z=-3.74, p<0.001). In the consensus condition the odds of having an argument from expertise were 290% higher in the U.S. compared to Japan.

*Complementary data.*

The full coding for the arguments was as follows:

*No argument* = No answer, or completely irrelevant answer.

*Perceptual argument* = Argument based on properties of the lines (e.g. “the three longest lines in 'A' seem to equal most of the smaller segments in 'B'”)

*Booster* = Argument based on personal attributes or motivation of the participant (indicating certainty or competence for instance), or simple restatement of the answer (e.g. “They are very close, but I believe that A is a bit longer than B,” or “I've answered most of these correctly so this is the correct answer”).

*Argument from expertise* = Argument that includes a mention of the information provided to the participant from the various sources (e.g. “Previous participants who have gotten most, if not all, questions similar to this have answered the same,” or “The person before me was always correct, so I just copied them”).

*Disclaimer* = Disclaimers, arguments that aim at making the answer less, rather than more, likely to be accepted (e.g. “I haven't guessed a lot of these correctly so far, so use your own best judgment. Good luck!”)

Figure S1 presents the full break-down of argument types as a function of condition and country.

*Figure S1.* Proportion of argument type as a function of condition and country, for participants who accepted the correct answer through deference to the expert.

**Experiment 2**

*Complementary analysis.*

This analysis presents all the data together, instead of analyzing the Confidence condition separately, as in the main text.

We used a binary variable (convinced or not by expertise) to analyse the results of a three-way interaction between country (U.S. and Japan), experiment (1 and 2) and condition (Competence, Consensus and Confidence). We found a significant interaction between country, experimental condition, and experiment. In the US, there was no difference between the three experimental conditions in the two experiments (not reported here in full) but we found an overall decrease in the influence of expertise between experience and description between the first and second experiment (GLM, β=-0.77, SE=0.33, z=-2.36, p=0.02). Similarly, in Japan, we found a similar decrease in the influence of expertise but this effect depended on condition, with a larger decrease in the consensus (GLM, β=-2.84, SE=0.38, z=-7.50, p<0.001) and competence condition (GLM, β=-2.11, SE=0.38, z=-5.64, p<0.001) and a non significant change in the confidence condition (GLM, β=-0.27, SE=0.32, z=-0.84, p=0.40).
