# Supplementary material for: Obstacles to the spread of unintuitive beliefs
Source: Evol Hum Sci. 2019 Oct 14;1:e10. doi: 10.1017/ehs.2019.10 (PMC10427286; doi:10.1017/ehs.2019.10)
Supplement: Supplementary file 1 [file S2513843X19000100sup001.zip › S2513843X19000100sup001/title page.docx]

OBSTACLES TO THE SPREAD OF COUNTER-INTUITIVE BELIEFS

Mercier, Hugo

Institut Jean Nicod, Département d’études cognitives, ENS, EHESS, PSL University, CNRS, Paris France [hugo.mercier@gmail.com](mailto:hugo.mercier@gmail.com) orcid.org/0000-0002-0575-7913

Corresponding author

Majima, Yoshimasa

Hokusei Gakuen University, Sapporo, Japan

Claidière, Nicolas

Aix Marseille Université, CNRS, LPC UMR 7290, 13331, Marseille, France

Léone, Jessica

Institut des Sciences Cognitives Marc Jeannerod, UMR 5304, CNRS & Université de Lyon, Bron, France
