# Supplementary figures and images for: Obstacles to the spread of unintuitive beliefs
Source: Evol Hum Sci. 2019 Oct 14;1:e10. doi: 10.1017/ehs.2019.10 (PMC10427286; doi:10.1017/ehs.2019.10)

## PAIR 1

**A**

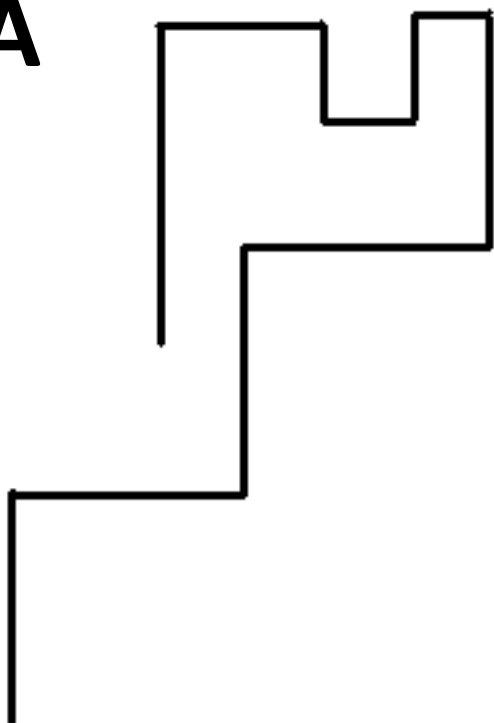

# B

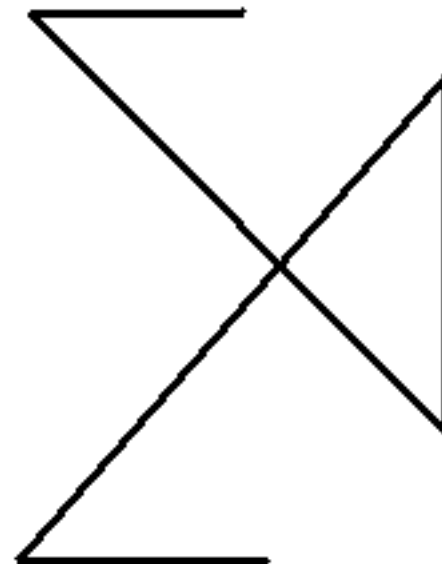

## PAIR 2

**A**

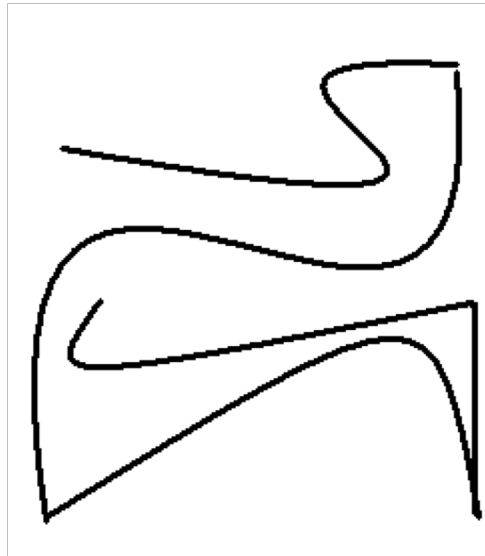

**B**

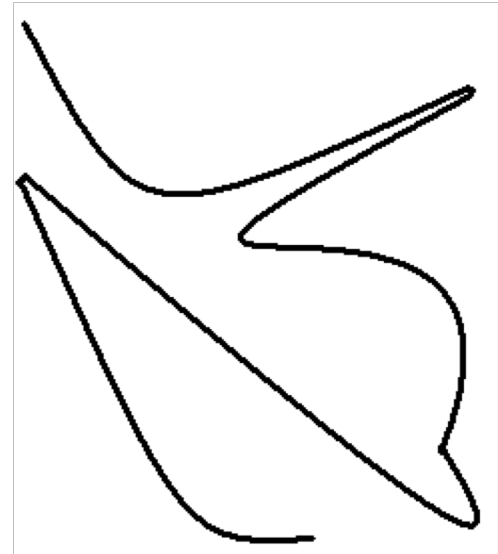

# PAIR 3

**A**

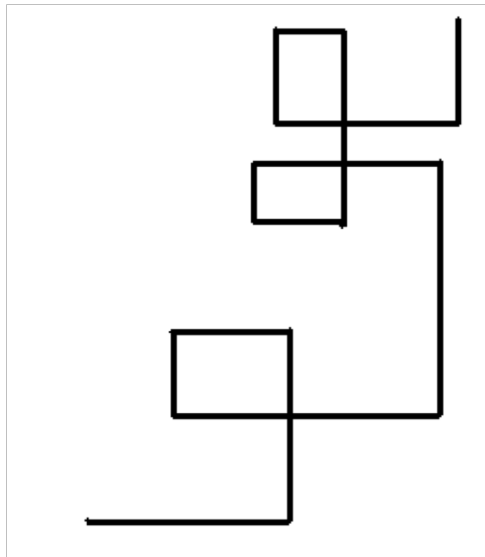

**B**

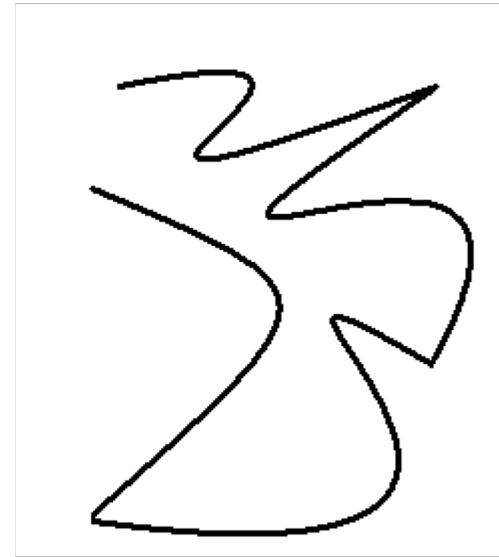

## PAIR 4

**A**

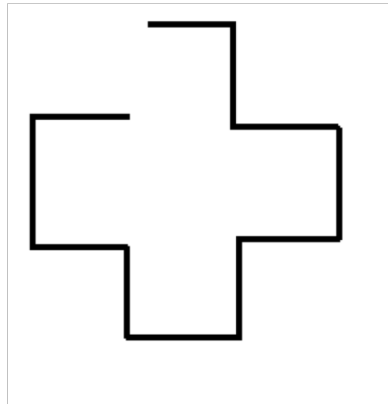

**B**

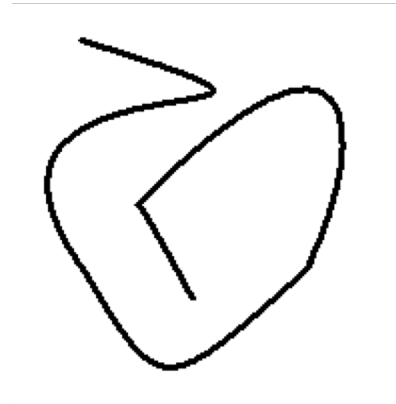

## PAIR 5

**A**

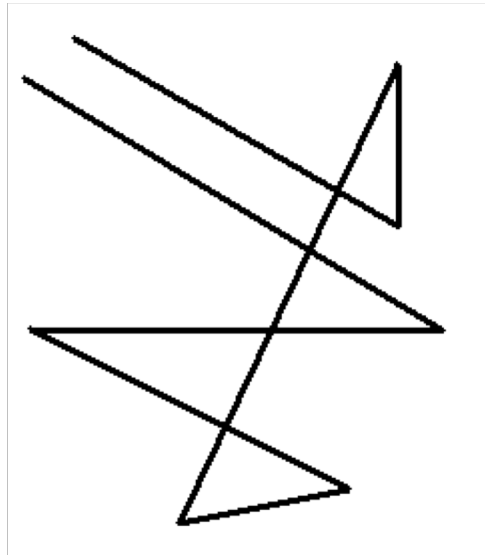

**B**

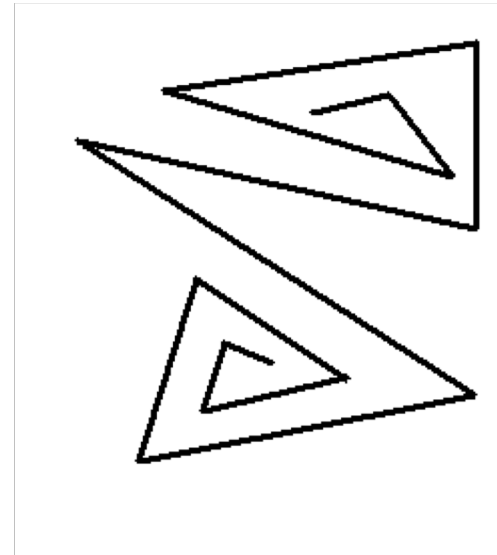

## PAIR 6

**A**

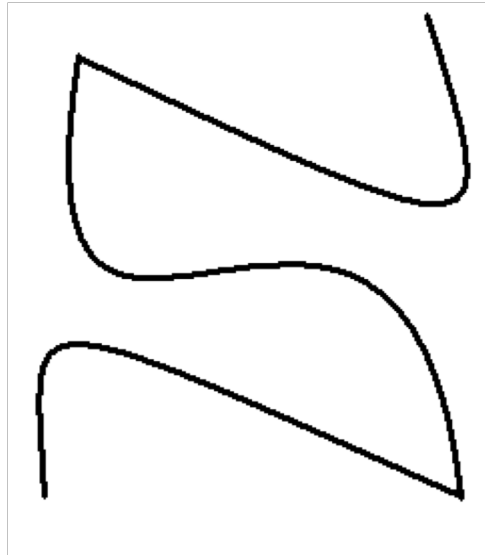

**B**

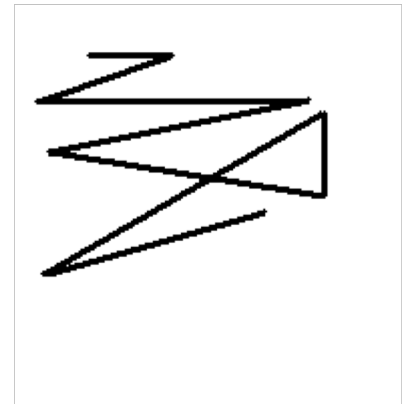

## PAIR 7

**A**

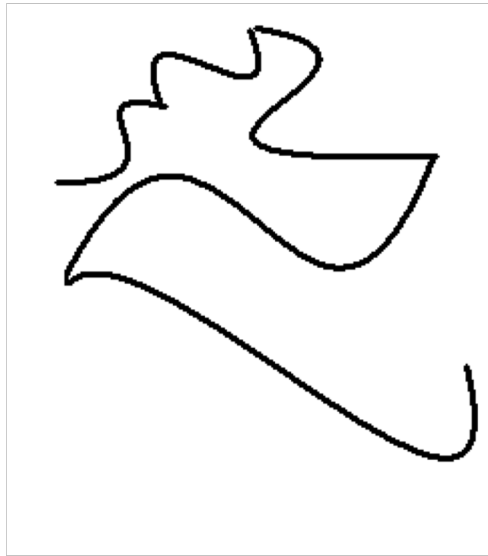

**B**

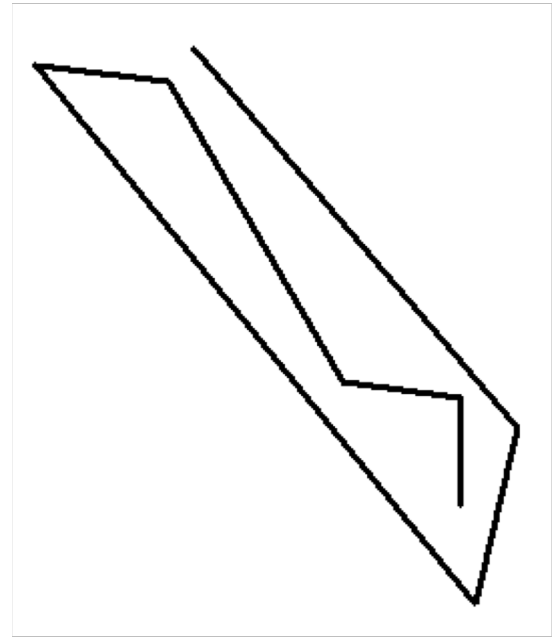

## PAIR 8

**A**

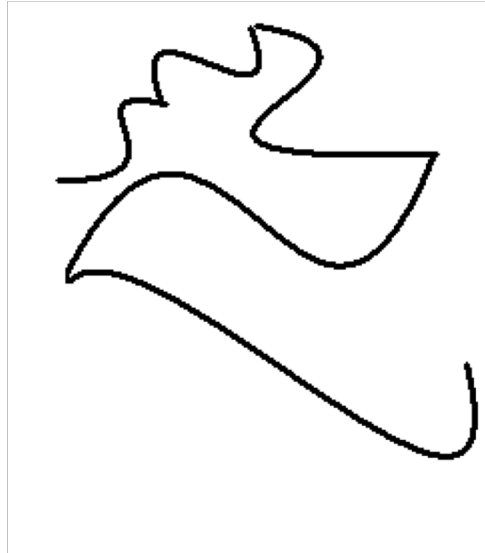

**B**

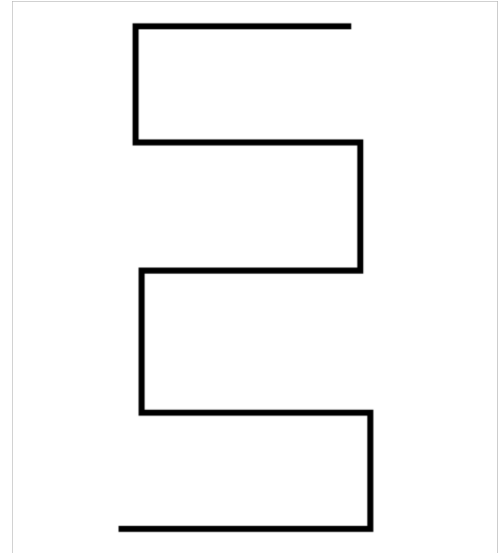

## PAIR 9

**A**

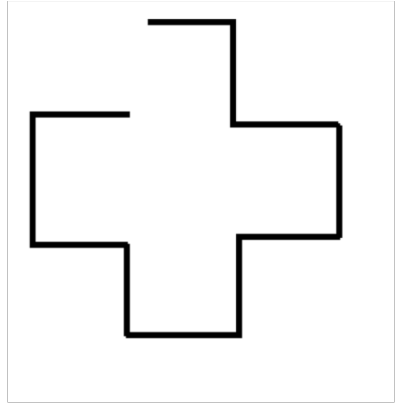

**B**

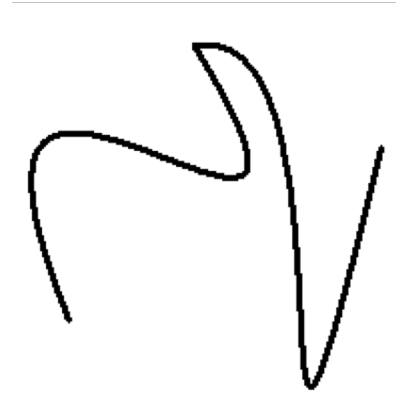

# PAIR 10

**A**

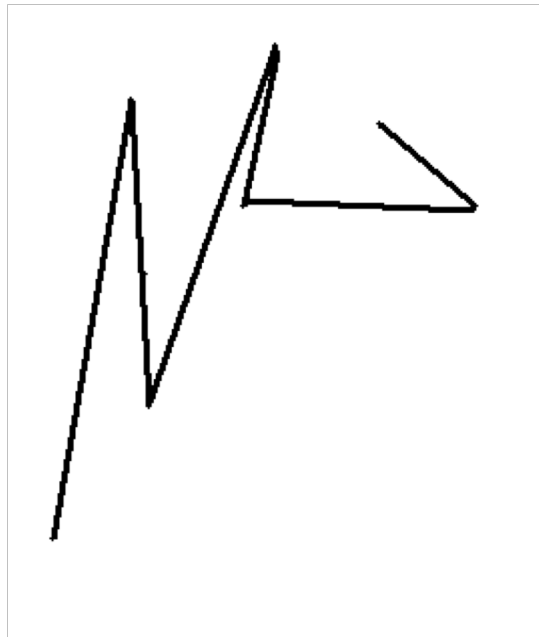

**B**

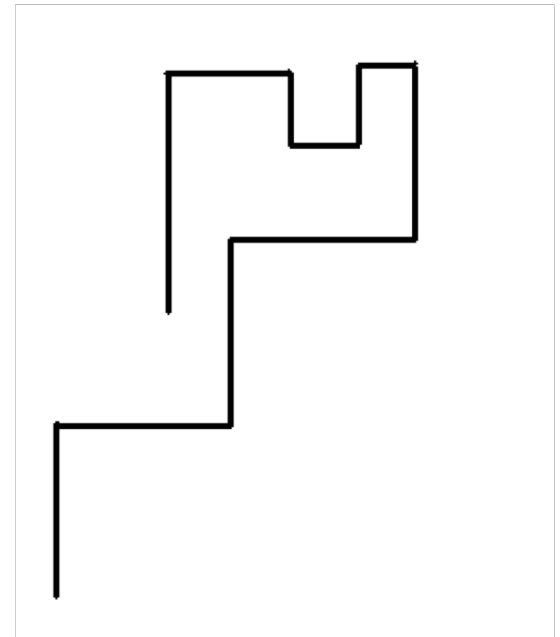

Supplement: Supplementary file 1 [file S2513843X19000100sup001.zip › S2513843X19000100sup001/ESM stimuli.pdf]
